# Clinical evidence of the link between gut microbiome and myalgic encephalomyelitis/chronic fatigue syndrome: a retrospective review

**DOI:** 10.1186/s40001-024-01747-1

**Published:** 2024-03-01

**Authors:** Jing-Hua Wang, Yujin Choi, Jin-Seok Lee, Seung-Ju Hwang, Jiyeon Gu, Chang-Gue Son

**Affiliations:** 1 Institute of Bioscience & Integrative Medicine, Daejeon University, 75, Daedeok-Daero 176, Seo-gu, Daejeon, 35235 Republic of Korea; 2 Department of Internal Medicine, College of Korean Medicine, Se-Myung University, Jecheon-si, 27136 Republic of Korea

**Keywords:** Chronic fatigue syndrome, Myalgic encephalomyelitis, Etiology, Gut microbiota, Metabolites

## Abstract

Myalgic Encephalomyelitis/Chronic Fatigue Syndrome (ME/CFS) is a heterogeneous disorder with elusive causes, but most likely because of clinical and other biological factors. As a vital environmental factor, the gut microbiome is increasingly emphasized in various refractory diseases including ME/CFS. The present study is aimed to enhance our understanding of the relationship between the gut microbiome and ME/CFS through data analysis of various clinical studies. We conducted a literature search in four databases (PubMed, Cochrane Library, Web of Science, and Google Scholar) until May 31, 2023. Our analysis encompassed 11 clinical studies with 553 ME/CFS patients and 480 healthy controls. A comparative analysis of meta data revealed a significant decrease in α-diversity and a noticeable change in β-diversity in the gut microbiome of ME/CFS patients compared to healthy controls. The notable ratio of Firmicutes and Bacteroides was 2.3 times decreased, and also, there was a significant reduction in the production of microbial metabolites such as acetate, butyrate, isobutyrate, and some amino acids (alanine, serine, and hypoxanthine) observed in ME/CFS patients. The lack of comparison under similar conditions with various standardized analytical methods has impeded the optimal calculation of results in ME/CFS patients and healthy controls. This review provides a comprehensive overview of the recent advancements in understanding the role of the gut microbiome in ME/CFS patients. Additionally, we have also discussed the potentials of using microbiome-related interventions and associated challenges to alleviate ME/CFS.

**Supplementary Information:**

The online version contains supplementary material available at 10.1186/s40001-024-01747-1.

## Introduction

Myalgic encephalomyelitis/chronic fatigue syndrome (ME/CFS) is a representative heterogeneous disease, with a prevalence of 0.89% worldwide and 0.77% in Korea [1, 2]. Individuals with ME/CFS suffer from unexplained severe fatigue lasting over six months, with key symptoms of excessive post-exertional malaise (PEM), unrefreshing sleep, and cognitive problems or orthostatic intolerance [3]. Epidemiological data from the Centers for Disease Control and Prevention (CDC) estimate, there are 0.84 to 2.5 million ME/CFS patients in the US, with approximately 25% homebound or bedridden [4].

While the underlying etiology of ME/CFS remains identified, but many debilitating circumstances trigger this disease including viral infection, immune dysfunction, neuroendocrine imbalance, genetic predisposition, psychological factors, and more [5, 6]. However, the true cause of ME/CFS pathophysiology has not been established yet, which has raised the difficulty to understand the disease objectives and determine the appropriate diagnosis and therapeutics [7]. Recently, the extensive research on chronic metabolic diseases, psychiatric disorders, and ME/CFS has revealed the potential involvement of gut microbiomes’ abnormal functionality in these disorders [8, 9]. Researchers have attempted to discover meaningful clues about ME/CFS through the human gut microbiome and its metabolites [10, 11]. In the clinic, a significant number of ME/CFS patients, ranging from 38% [12] to 42% [13], also experience irritable bowel syndrome (IBS), and over 70% of ME/CFS patients report various gastrointestinal disturbances [14], suggesting a potential link between disrupted gut microbiome and ME/CFS pathophysiology.

The perturbed gut microbiome is known to influence the brain function through the dysregulated gut-brain axis [15, 16]. Gut microbiome imbalance is associated with increased gut permeability leading to exacerbate the inflammation gradually in multiple organ systems, including the brain [17]. Many studies have also reported a link between gut microbiome dysbiosis and depression [18]. In light of the recent understanding of ME/CFS as a multisystem neuroimmune disease, the variation in gut microbiome derived metabolites might contribute to ME/CFS [19]. However, numerous essential questions remain unanswered, including whether ME/CFS patients consistently show alterations in their gut microbiome and its related metabolites, and if so, how these alterations interact with ME/CFS progression?

To address these questions, we conducted a comprehensive analysis based on recent clinical reports comparing alterations in the gut microbiome and its associated metabolites in ME/CFS patients to healthy controls.

## Methods

### Protocol registration

The current study protocol was registered on PROSPERO (International Prospective Register of Systematic Reviews) with the registered number of CRD42023445298. The protocol is available at the following link: [https://www.crd.york.ac.uk/prospero/display_record.php?ID=CRD42023445298].

### Literature searching strategy

The systematic review was performed in adherence to the guidelines provided by the PRISMA framework [20]. The relevant literature was surveyed through four well-known databases of biomedical literature until the data of 31st May 2023, including PubMed (www.ncbi.nlm.nih.gov/pubmed), Cochrane library (www.cochranelibrary.com), Web of Science (www.webofscience.com), and Google Scholar (scholar.google.com) with the combinations of the following key terms: (“microbiota OR microbiome” combined with “chronic fatigue syndrome OR myalgic encephalomyelitis”; *title/abstract*).

### Inclusion and exclusion criteria

We selected clinical literature with healthy control groups based on their inclusion of gut microbiome-associated analysis directly related to ME/CFS. Exclusions comprised reviews, non-clinical studies, studies lacking control groups, those focusing on the oral microbiome without relevance to ME/CFS, repetitive studies, those without available full texts, and non-English papers.

### Review process and data extraction

Two authors (J.-H. Wang and Y. Choi) conducted the search and selection of eligible articles based on the aforementioned criteria. Manual screening was employed to remove any duplicate papers. The selected articles provided data on various parameters including the number of participants, average age, race/ethnicity, publication year, country, sample type, method of microbiome determination, instruments used, bioinformatics tools applied, diagnostic criteria of ME/CFS, fatigue assessment, gastrointestinal complications, α and β diversity, microbial metabolites, and bacterial abundance. Information was extracted from the text, figures, and supplementary materials of each included paper. In cases where only graphical data were available, the Web-Plot-Digitizer app (version 4.6) was utilized to extract relevant parameters from the graphs (https://apps.automeris.io/wpd/).

### Assessments of study quality, publication bias, and heterogeneity of outcome

The quality assessment of each study was conducted using the six domains of the Cochrane Collaboration’s tool, which include random sequence generation (selection bias), allocation concealment (selection bias), blinding of participants and personnel (performance bias), blinding of outcome assessment (detection bias), incomplete outcome data (attrition bias), selective reporting (reporting bias). The risk of bias for each domain was evaluated and categorized as ‘low’, ‘unclear’, or ‘high’. The comprehensive assessment results are shown in Additional file 2: Figure S1. We also assessed the potential for publication bias in a meta-analysis using funnel plots and Egger’s test (Additional file 3: Figure S2). In assessing the heterogeneity of studies, the *I*^2^ value was utilized to describe the probability of total variation across studies, stemming from heterogeneity rather than mere chance or random error [21]. An *I*^2^ value of 50% indicates considerable heterogeneity resulting from actual differences in study populations, protocols, interventions, and outcomes.

### Statistical analysis

In the present study, the meta-analysis including forest plots was conducted for data of gut microbiome α diversity using RevMan 5.4 statistical software from Cochrane (Oxford, UK). Results were expressed as standardized mean difference (SMD) with 95% confidence intervals (CI). Based on the assessment of heterogeneity using *I*^2^ statistics, we applied a random-effects model for cases with 50% or higher heterogeneity. Statistical significance was defined by *p*-values less than 0.05.

## Results

### Descriptions of included studies

**Fig. 1:**
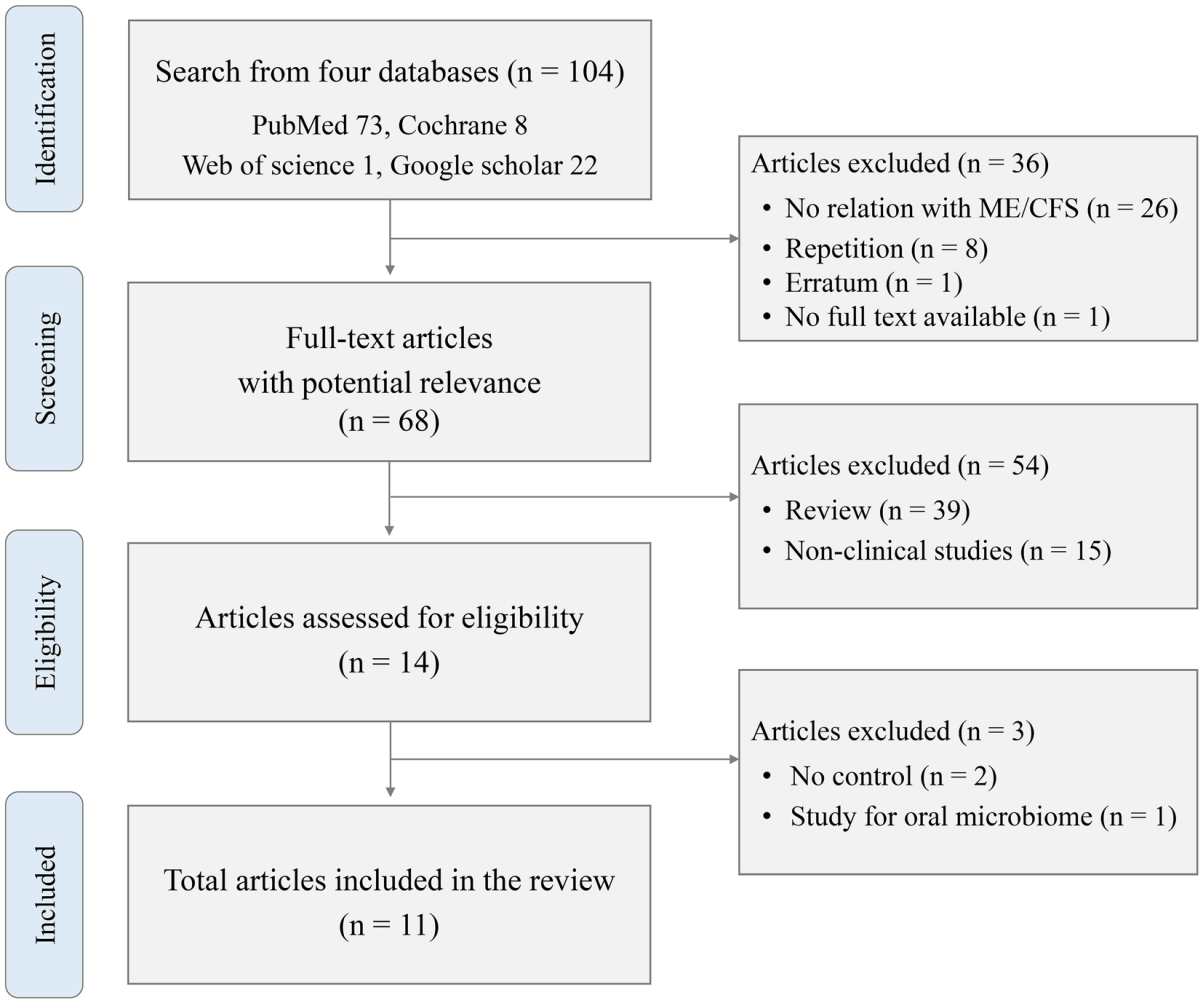
Assessment of the clinical research to be included in this study for further analysis of Gut microbiome during ME/CFS

From four electronic databases, we identified a total of 104 studies; 73 from PubMed, eight from Cochrane, one from Web of Science, and 22 from Google Scholar. Out of these, 93 studies were excluded for various reasons, as follows: eight duplicates, 26 unrelated to ME/CFS, one erratum, one lacked full text, 39 review articles, 15 non-clinical studies, two without a healthy control, and one focused only on the oral microbiome (Fig. 1). Eventually, we selected 11 clinical studies (listed in Additional file 1: Table S1 and labeled as I to XI) that met the inclusion and exclusion criteria for the present review. The selected studies were conducted in six countries (63.6% in USA) across four continents, and were published within the past decade (Table 1).

**Table 1:** Summary of characteristics of included 11 studies

Items	ME/CFS			Healthy control			Total
Number of participants (%) (Mean ± SD)	553 (100%) (52 ± 42)			480 (100%) (45 ± 28)			1033 (100%) (48 ± 35)
Male	124 (22.4%)			118 (24.6%)			242 (23.4%)
Female	429 (77.6%)			362 (75.4%)			791 (76.6%)
Age (Mean ± SD)^a^	45.0 ± 6.7			43.9 ± 6.8			44.5 ± 6.9
BMI (kg/m^2^)	24.3 ± 2.2			24.5 ± 1.8			24.4 ± 1.9
Diagnostic criteria,	N. of study						Percentage
Fukuda	9			N/A			69.2%
Canadian	3			N/A			23.1%
ICC	1			N/A			17.7%
Participants with GI disorder							
IBS (4 studies)	177/273 (64.8%)			10/280 (3.6%)			187/553 (33.8%)
GID (4 studies)	34/98 (34.7%)			17/87 (19.5%)			51/185 (27.6%)
N/A (3)	182			113			295
Gut microbiome detection methods (Number of study, %)							
16 s/18 s rRNA (7, 63.6%)	Shotgun (3, 27.3%)				Culture (1, 9.1%)		
Instruments, Number of study (%)							
Illumina MiSeq	Illumina HiSeq		Roche 454			MALDI-TOF MS	
5 (45.5%);	3 (27.3%)		2 (18.2%)			1 (9.1%)	
Gut microbiota analysis tools, N. of study (%)							
QIIME 1/2	R	Mothur		MBT			RDP Classifier
5 (45.4%)	2 (18.2%)	2 (18.2%)		1 (9.1%)			1 (9.1%)
Publication Year, Number of study (%)							
2013–2020 (7 studies, 63.6%)			2021–2023 May (4 studies, 36.4%)				
Continents, Number of study (%)							
North America	Europe		Oceania				Asia
7 (63.6%)	2 (18.2%)		1 (9.1%)				1 (9.1%)

*ME/CFS* Myalgic encephalomyelitis/chronic fatigue syndrome, *N/A* Not available, *IBS* irritable bowel syndrome, *CCC* Canadian consensus criteria, *ICC* international consensus criteria, *GID* gastrointestinal disturbances, *QIIME* Quantitative insights into microbial ecology, *MBT* MALDI Biotyper, *RDP* Ribosomal database project, *MALDI-TOF MS* Matrix-assisted laser desorption ionization-time of flight mass spectrometry

^a^Only 1 study use median of age was excluded

### Characteristics of the participants

A total 1,033 participants (23.4% of male and 76.6% of female) consisting of 480 healthy (46.5%) and 553 ME/CFS (53.5%) subjects were enrolled in 11 studies from six countries, the average age of all subjects was around 44.5 years old (healthy control 43.9 ± 6.8, ME/CFS 45.0 ± 6.7). The average body mass index (BMI) of all participants was below 25, as not notably differently between healthy control (24.5 ± 1.8) or ME/CFS status (24.3 ± 2.2), respectively. In the 11 studies reviewed, three case criteria for ME/CFS diagnosis were employed: six using Fukuda criteria and one using Canadian Consensus Criteria (CCC) exclusively, while two studies as a combination of Fukuda and CCC, and one study employing a combination of the Fukuda and the International Consensus Criteria (ICC), respectively. In terms of gastrointestinal complications, 58.3% of ME/CFS patients experienced gastrointestinal dysfunction in contrast to only 9.2% of healthy subjects (Table 1).

All 11 selected studies analyzed the gut microbiome using Next-Generation Sequencing (NGS, 10 studies) or in vitro anaerobic culture method (one study), all focused on fecal samples. However, only five studies performed comparative analyses of gut microbial metabolites in urine, serum/plasma or feces. All selected studies were conducted in six countries across four continents, and published within the past decade (Table 1). In addition, eight out of the 11 selected studies mentioned that subjects who had taken any type of antibiotics in the prior two or four weeks were excluded.

### Diminished gut microbiome α-diversity in ME/CFS patients

**Fig. 2:**
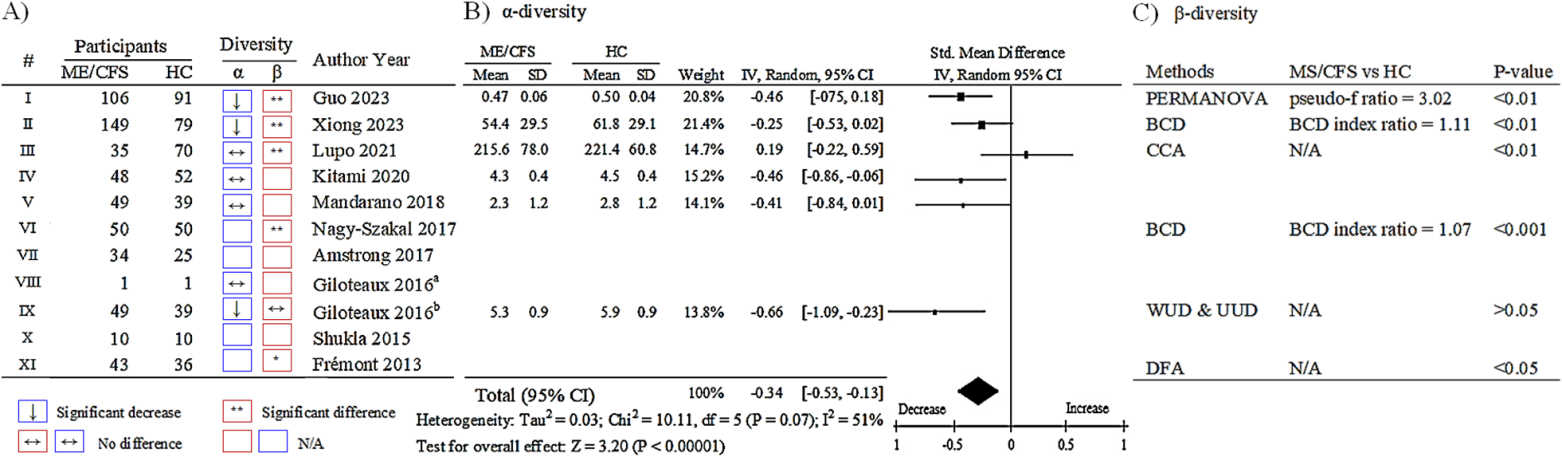
Alterations of α- and β-diversity of gut microbiome in ME/CFS patients **A** Outline of gut microbiome α- and β-diversity change from the 11 selected studies, denoted by Roman numerals I to XI. **B** Forest plots summarizing alpha-diversity of gut microbiome using the random-effects meta-analysis model. **C** List of gut microbiome β-diversity in gut microbiome of ME/CFS patients versus healthy controls. *CI* confidence interval, *df* degrees of freedom; *I*^2^, I-square heterogeneity statistic, *ME/CFS* Myalgic encephalomyelitis/chronic fatigue syndrome, *HC* healthy control, *N/A* non-available, *CCA* Canonical correspondence analysis, *BCD* Bray–curtis dissimilarity, *WUD* Weighted UniFrac distance, *UUD* Unweighted UniFrac distance; *DFA* Discriminant function analysis. *P < 0.05 and **P < 0.01 ME/CFS patients compared to the healthy controls

Among 11 studies, seven reported the α-diversity of gut microbiome, with a significant reduction in 3 studies, and no significant difference in 4 studies, incorporating various evaluation methods such as Shannon, Chao 1, observed species, and Pielou’s index (Fig. 2A). One of these studies mentioned only the lack of substantial difference without reporting the quantitative information. Therefore, we conducted a meta-analysis of α-diversity values from six studies. Considering *I*^2^ = 51% heterogeneity (*P* > 0.07), we applied a random-effects model to calculate the standardized mean difference (SMD). The result indicated a significant 34% decrease in α-diversity of gut microbiome in ME/CFS patients compared to healthy controls (P < 0.00001, Fig. 2B).

### Alterations of gut microbiome β-diversity in ME/CFS patients

Among 11 studies, six studies reported the β-diversity of gut microbiome (Fig. 2A). Expect for one study that showed no noticeable alteration in the β-diversity of the gut microbiome (*P* > 0.05), the remaining five studies consistently indicated a significant dissimilarity in the overall structure of the gut microbiome in ME/CFS patients compared to healthy controls (*P* < 0.05, 0.01 or 0.001, Fig. 2A, C).

### Taxonomic changes in gut microbiome of ME/CFS patients

**Fig. 3:**
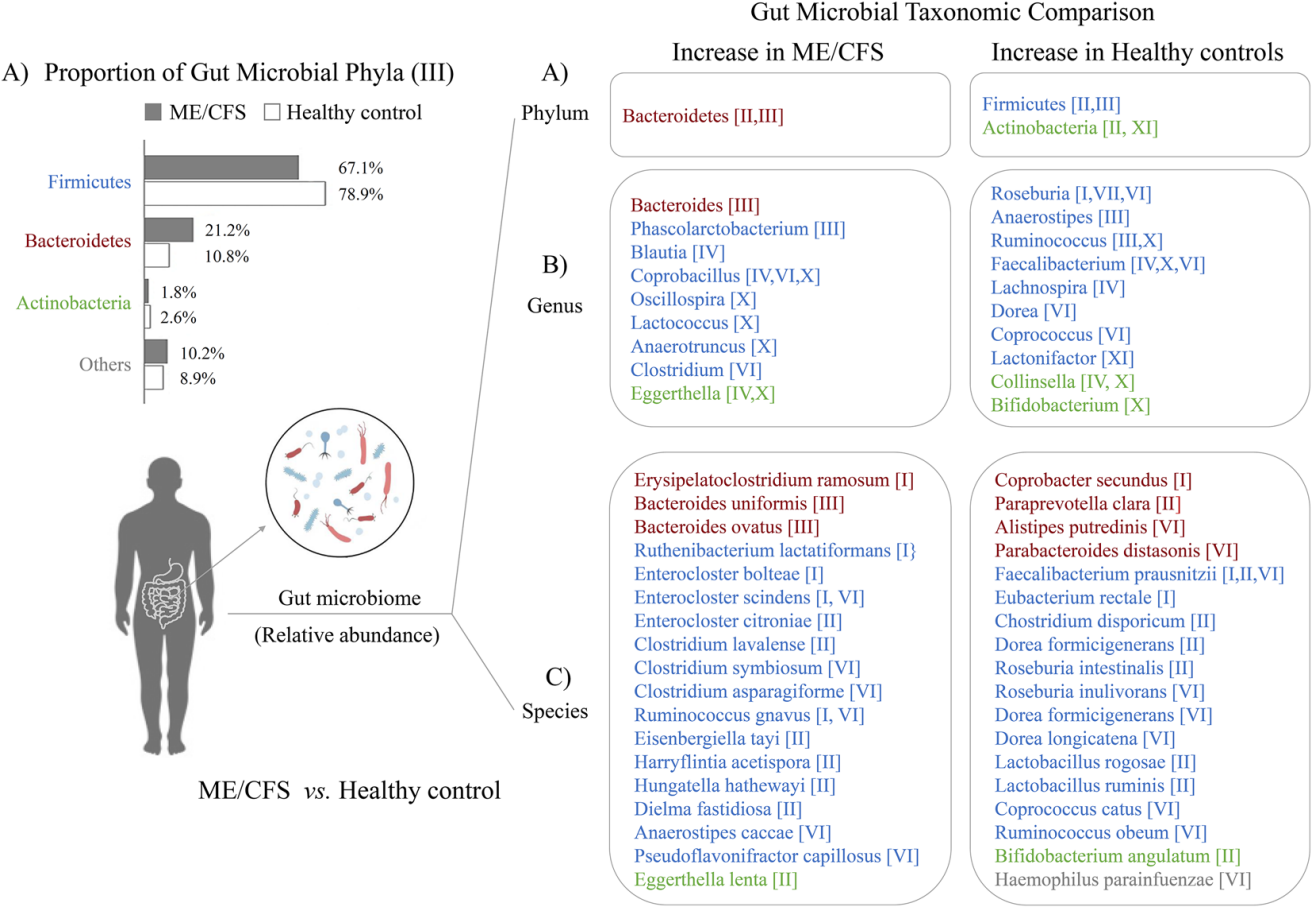
Remarkable Taxonomic Changes in the Gut Microbiome of ME/CFS Patients We listed significant differences (*P* < 0.05) in the relative abundance of the gut microbiome between ME/CFS and healthy control, including taxonomic classifications at the phylum **A**, genus **B**, and species **C** levels. Detailed reference information is available in Additional file 1: Table S1. **P < 0.01 ME/CFS patients compared to the healthy controls

All 11 studies compared the taxonomic changes in the gut microbiome, by utilizing 16 s rRNA sequencing (six studies), whole-genome shotgun metagenomic sequencing (three studies), 18 s rRNA sequencing (one study), or anaerobic culture (one study), respectively. We herein summarized the alterations of gut microbiome in terms of three levels of taxonomic classification, from 10 studies except anaerobic culture-derived data (Fig. 3A–C).

The detailed alterations of gut microbiome by three levels of taxonomic classification from the 10 studies were summarized. At the phylum level, ME/CFS patients showed a significant increase in Bacteroidetes, along with noticeable reductions in Firmicutes compared to healthy controls (*P *< 0.01, Fig. 3A). Interestingly, some potentially beneficial genera such as *Bifidobacterium*, *Roseburia*, and *Faecalibacterium* display a notable decrease in CFS patients compared to healthy controls (*P* < 0.05 or *P* < 0.01, Fig. 3C). While Firmicutes are notably reduced, certain genera belonging to Firmicutes, such as *Phascolarctobacterium, Blautia, Coprobacillus, Oscillospira, Lactococus, Anaerotruncus,* are increased significantly (*P* < 0.05 or *P* < 0.01, Fig. 3B).

Regarding species-level differences, only one study (II, Additional file 1: Table S1) provided the complete raw data on detected gut microbiome species (64 ± 14 species in 78 healthy controls and 58 ± 16 species in 146 ME/CFS patients). Additionally, 4 studies showed a significant increase of some opportunistic pathogenic bacterial species among ME/CFS patients, including *Erysipelatoclostridium ramosum, Enterocloster citroniae, Hungatella hathewayi, Eggerthella lenta,* etc. Conversely, some lactic acid bacteria and other beneficial species notably decreased in ME/CFS patients, such as *Faecalibacterium prausnitzii, Bifidobacterium angulatum, Lactobacillus ruminis, Roseburia intestinalis *etc*.* (Fig. 3C).

### Alteration of potential gut microbiome related metabolites in ME/CFS patients

**Fig. 4:**
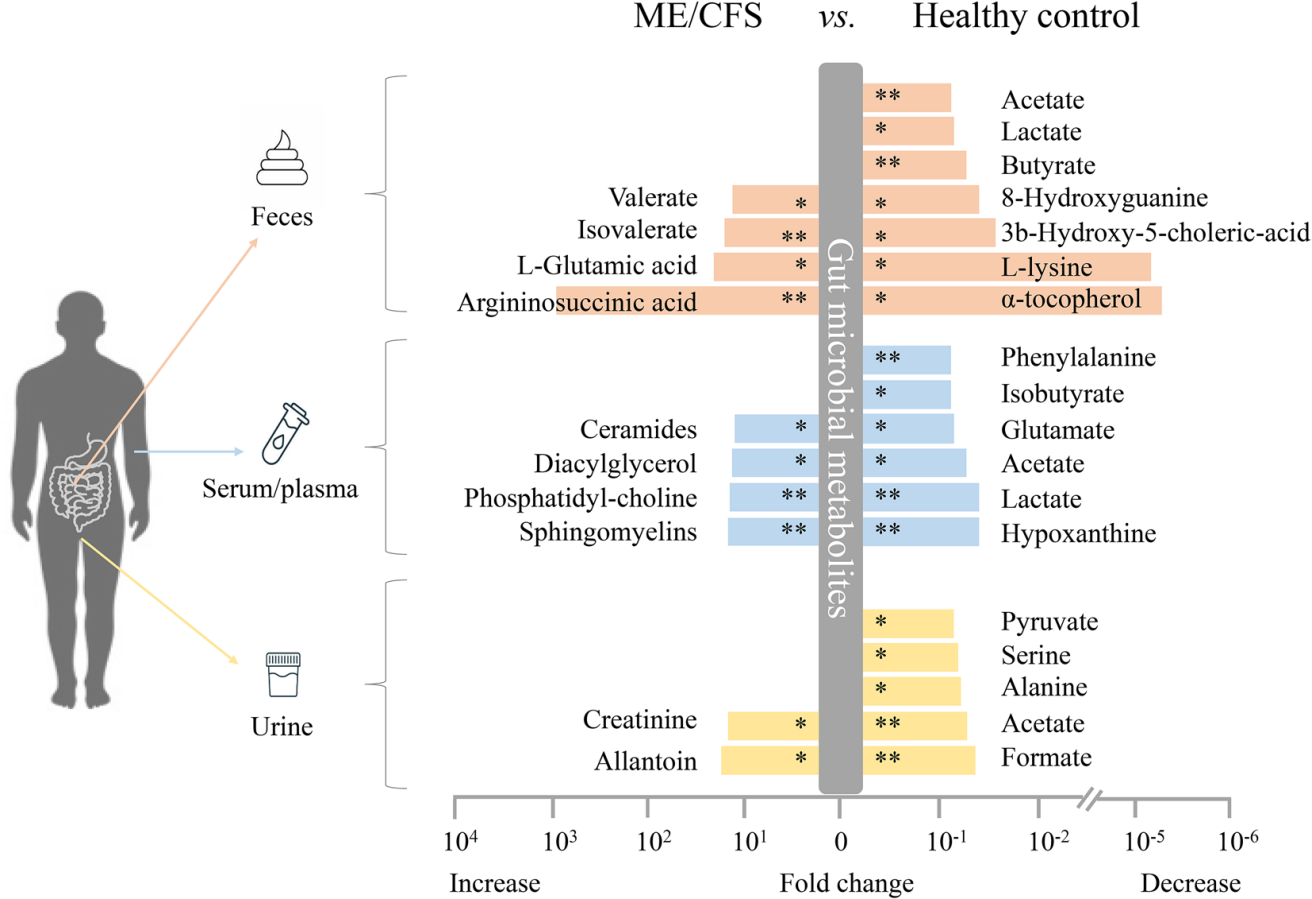
Microbial metabolites alteration in ME/CFS patients A summary of the noteworthy alterations in microbiome-related metabolites in ME/CFS patients’ feces, serum/plasma and urine compared to healthy control. ME/CFS: Myalgic Encephalomyelitis/Chronic Fatigue Syndrome. *P < 0.05 and **P < 0.01 ME/CFS patients compared to the healthy controls

A total five studies have reported potential differences in metabolites between ME/CFS patients and healthy controls, using feces (four studies), serum/plasma (three studies), and/or urine (one study). Particularly, both short-chain fatty acids (SCFA, like butyrate, acetate) and branched SCFA such as isobutyrate were significantly reduced in fecal, blood, or urine of ME/CFS patients. Remarkably, an extensive reduction of serum α-tocopherol (Vitamin E, *P* < 0.05), a typical antioxidant vitamin that can be potentially metabolized by certain gut microbes, was observed in ME/CFS patients compared to healthy controls. Besides, the notable differences in other metabolites, mainly including amino acids and lipid molecules directly or indirectly influenced by gut microbiome also observed (Fig. 4).

## Discussion

Recently, increasing studies have stressed the importance of gut microbiome in various clinical conditions, including irritable bowel syndrome [22], and psychiatric disorders [23]. It’s worth noting that typical symptoms in ME/CFS, such as digestive disorders, depression, cognitive issues, and even fatigue itself, are also deemed to be closely associated with gut microbiome dysbiosis [19, 24, 25]. To uncover clues of the pathogenesis of ME/CFS, we herein comprehensively analyzed the latest clinical data concerning the gut microbiome and related metabolites.

Although the number of included studies were less than expected, we ultimately selected 11 studies that met our study criteria, encompassing 553 individuals with ME/CFS and 480 healthy controls. In the basic analyses of dissimilarities among different ecosystems, the comparisons of α-diversity and β-diversity are commonly employed [26]. From six available studies (436 ME/CFS patients and 370 healthy controls), we observed a significant reduction of the α-diversity of the gut microbiome in ME/CFS patients compared to the healthy controls (Fig. 2A, B). Although it’s not definitive, many scientists believe that the healthy individual typically harbors approximately 500 to 1000 difference species of microbiome within the human gut [27]. Our data revealed a reduction of around 34% of gut microbiome species number in ME/CFS patients compared with healthy controls (*P* < 0.00001). In general, healthy individuals harbor a greater variety of different microbiome species in gut, and this diversity contributes significantly to the overall health of the host [28]. However, it is noteworthy that reduced α-diversity has been commonly observed not only in ME/CFS but also in many other pathological conditions and diseases, such as obesity, diabetes, inflammatory bowel diseases (IBD), and mental health disorders [29–33]. Moreover, aging is associated with changes in the gut microbiome and a reduction in gut microbial α-diversity is evidently observed in older individuals [34, 35]. Nevertheless, our study showed that the participants with ME/CFS and healthy controls were recruited within similar average ages and ranges (ME/CFS: 45.0 ± 6.7 years old and healthy controls: 43.9 ± 6.8 years old).

On the other hand, in contrast to α-diversity, the β-diversity indicates the extent of the difference in the composition of gut microbial species between two groups [36]. When we analyzed the β-diversity from six available studies, except one study with no significant difference (*P* > 0.05), five studies consistently confirmed a notable distinction in the structural similarity of the gut microbiome between ME/CFS and healthy individuals (*P *< 0.05, 0.01 or 0.001, Fig. 2A, C). Similar to α-diversity, the obvious dissimilarity of the gut microbial communities (β-diversity) is also not exclusive to ME/CFS but is also observed in conditions such as IBD, metabolic diseases, autoimmune diseases, neurological disorders [37–40]. The further studies, however, are required, to directly compare the β-diversity of gut microbiome between ME/CFS and other similar diseases, like fibromyalgia, long COVID, and autoimmune diseases, in the future. In general, the significant reduction in species diversity and substantial alteration in the composition of gut microbiome can impact host metabolism, consequently resulting in a series of changes in metabolites [41]. One research revealed that a significant change in gut microbiome α-diversity was observed in short-term ME/CFS patients, while obvious metabolic and clinical aberrations in long-term ME/CFS patients [11].

When we conducted an analysis of the overall changes in gut microbiome taxonomy and related metabolites from three sources, significant changes in the relative abundance of gut microbiome phyla, genera, and species were observed (Fig. 3). Simultaneously, notable alterations were found in the related metabolites, including 11 in feces, 10 in serum/plasma, and seven in urine (Fig. 4). Among these alterations, a significant decline in serum butyrate levels were found in ME/CFS patients [19]. We also observed a decrease in butyrate-producing bacteria among ME/CFS patients, which has been confirmed by several studies involving genus/species like *Ruminococcus*, *Faecalibacterium prausnitzii*, and *Eubacterium rectale* [11, 13, 19, 42]. Moreover, the abundance of these species were negatively correlated with the severity of fatigue symptoms in ME/CFS patients [19]. Besides, noticeable decreases in other bacteria-produced metabolites, which serve as energy sources (such as acetate and isovalerate) and exhibit antimicrobial properties (such as lactate and benzoate), were observed in the ME/CFS patients as well [43]. These changes were positively correlated with *Clostridium*, but negatively correlated with *Bacteroides*. [43]. In addition, gut microbiome-derived sphingolipids have been demonstrated to alter host lipid metabolism [44]. Specifically, *Bacteroides spp.* with serine palmitoyltransferase (SPT) gene have the ability to produce sphingolipids [44]. Therefore, this could partially elucidate the elevated levels of serum lipid profile and high Bacteroidetes were appeared simultaneously in long-term ME/CFS patients [11]. Numerous studies revealed the potential role of vitamin E in improving mental health, particularly cognitive function [45, 46]. Whereas, certain gut microbes, like *Bacteroides* and *Clostridium,* are considered to possess the potential ability to metabolize vitamin E in the intestinal tract [47, 48]. Hence, we suggest that the cognitive issues observed in ME/CFS patients may be associated to specific changes in the gut microbiome and a significant reduction in vitamin E levels. Tryptophan, serving as a precursor for serotonin, can be degraded by certain gut microbiome through various pathways, leading to the influence of serotonin levels and brain function [49] and even pathophysiology of ME/CFS [50]. Therefore, we suggest that exploring the alterations in gut microbiome composition and function could elucidate how tryptophan metabolism impacts serotonin levels, potentially contributing to several symptoms of ME/CFS, such as depression, unrefreshing sleep, as well as other pathological conditions. Regrettably, none of the 11 studies included in the current review conducted any analysis related to tryptophan metabolites.

Recently, the proposed causality or association between alterations in the gut microbiome and ME/CFS has become an intriguing subject for investigation [51]. A clinical analysis showed that patients with severe ME/CFS have excessive antibodies against flagellins, especially as a pathogen-associated molecular pattern (PAMP) from lachnospiraceae, compared to the healthy controls [42]. These findings suggested that the commensal microbiome may be directly involved in the pathogenic process of ME/CFS. Regarding the therapeutic aspect, a clinical trial demonstrated the efficacy of probiotics (*lactobacillus casei* strain Shirota and *bifidobacterium infantis* 35,624) administration in reducing anxiety and inflammatory biomarkers in ME/CFS patients [52]. An increasing number of clinicians believe that manipulating gut microbiome through fecal microbiome transplantation (FMT) might be an effective therapeutic for certain diseases including ME/CFS [53]. In fact, FMT using standardized live fecal microbiome (Rebyota^®^, Ferring Pharmaceuticals Inc., USA) has been initially approved by the U.S. Food and Drug Administration (FDA) to treat Clostridium difficile infection (CDI) in 2022 [54]. Besides, an oral standardized microbiome capsule (Vowst^™^, Seres Therapeutics, USA) also received FDA approval in April 2023 [55]. However, FMT failed to ameliorate the symptoms and health-related quality of life (QOL) in ME/CFS patients from a randomized, double-blinded, placebo-controlled clinical study [56]. These negative outcomes might be related to that conclusive evidence concerning whether the gut microbiome is one of the etiological agent for ME/CFS has not been established to date. Hence, the application of human fecal microbiome transplantation (FMT) into a germ-free or antibiotics-treated animal models could be considered a valuable approach for investigating the potential etiology and pathogenesis of ME/CFS.

As a well-known knowledge of female-dominant prevalence in ME/CFS [57], the current study indicated that 83% of the participants diagnosed with ME/CFS are female (Table 1). Actually, dissimilarity in the gut microbiome has been found between genders owing to genetic factors, hormonal influences and physiological variances [58]. However, the limitation of the present review is that none of the studies analyzed the gut microbiome differences as sex-specific. Regarding the methodology, 16 s rRNA sequencing has been the major technique for gut microbiome in the included ME/CFS studies. Nonetheless, we have observed an increasing number of researches switching to whole-genome shotgun sequencing to acquire more valuable information, driven in part by reduction in sequencing costs. Thus, variable methods and sequencing platform in the selected studies may hinder to draw firm conclusions. In addition, the gut microbiome generally includes not only bacteria but also archaea, fungi, viruses, protozoa, etc. However, the current review only focuses on gut bacteria due to the very few studies on ME/CFS that have explored aspects other than bacteria. Eventually, it is also a limitation that three out of 11 included studies did not mention the subjects’ condition of antibiotics use.

## Conclusions and future perspectives

In conclusion, our findings confirm a significant reduction in the gut microbiome species (α-diversity) among ME/CFS patients compared to healthy individuals. Moreover, the overall similarity in the structure of the gut microbiome (β-diversity) showed a notable difference between ME/CFS patients and healthy subjects. Although observable changes in certain gut microbiome and associated metabolites, particularly vitamin E, short chain fatty acids, have been partially identified, the precise mechanisms linking between ME/CFS and gut microbiome remain elusive thus far. Therefore, further research is essential to validate the causality and specificity of gut microbiome in ME/CFS. These investigations will offer valuable insights into the etiology, mechanism, diagnosis, treatment, prevention, and prognosis of ME/CFS.

### Supplementary Information

**Additional file 1: Table S1.** List of selected 11 clinical studies in the present review.

**Additional file 2: Figure S1.** Risk of bias summary.

**Additional file 3: Figure S2.** Funnel plot of included 6 studies for meta-analyzing gut microbiome α-diversity.

## Data Availability

The data that support the findings of this study are available within the article.

## References

[1] Lim EJ, Ahn YC, Jang ES, Lee SW, Lee SH, Son CG (2020). Systematic review and meta-analysis of the prevalence of chronic fatigue syndrome/myalgic encephalomyelitis (CFS/ME). J Transl Med.

[2] Lim EJ, Son CG (2021). Prevalence of chronic fatigue syndrome (CFS) in Korea and Japan: a meta-analysis. J Clin Med.

[3] Lim EJ, Son CG (2020). Review of case definitions for myalgic encephalomyelitis/chronic fatigue syndrome (ME/CFS). J Transl Med.

[4] Clayton EW (2015). Beyond myalgic encephalomyelitis/chronic fatigue syndrome: an IOM report on redefining an illness. JAMA.

[5] Wirth KJ, Löhn M (2023). Myalgic encephalomyelitis/chronic fatigue syndrome (ME/CFS) and comorbidities: linked by vascular pathomechanisms and vasoactive mediators?. Medicina.

[6] Ludwig B, Olbert E, Trimmel K, Seidel S, Rommer PS, Müller C, Struhal W, Berger T (2023). Myalgic encephalomyelitis/chronic fatigue syndrome: an overview of current evidence. Nervenarzt.

[7] Grach SL, Seltzer J, Chon TY, Ganesh R (2023). Diagnosis and management of myalgic encephalomyelitis/chronic fatigue syndrome. Mayo Clin Proc.

[8] Lim D-W, Wang J-H (2022). Gut microbiome: the interplay of an “invisible organ” with herbal medicine and its derived compounds in chronic metabolic disorders. In Int J Environ Res Publ Health.

[9] Boolani A, Gallivan KM, Ondrak KS, Christopher CJ, Castro HF, Campagna SR, Taylor CM, Luo M, Dowd SE, Smith ML (2022). Trait energy and fatigue may be connected to gut bacteria among young physically active adults: an exploratory study. Nutrients.

[10] Che X, Brydges CR, Yu Y, Price A, Joshi S, Roy A, Lee B, Barupal DK, Cheng A, Palmer DM (2022). Metabolomic evidence for peroxisomal dysfunction in myalgic encephalomyelitis/chronic fatigue syndrome. In Int J Mol Sci.

[11] Xiong R, Gunter C, Fleming E, Vernon SD, Bateman L, Unutmaz D, Oh J (2023). Multi-'omics of gut microbiome-host interactions in short- and long-term myalgic encephalomyelitis/chronic fatigue syndrome patients. Cell Host Microbe.

[12] Chu L, Valencia IJ, Garvert DW, Montoya JG (2019). Onset patterns and course of myalgic encephalomyelitis/chronic fatigue syndrome. Front Pediatr.

[13] Nagy-Szakal D, Williams BL, Mishra N, Che X, Lee B, Bateman L, Klimas NG, Komaroff AL, Levine S, Montoya JG (2017). Fecal metagenomic profiles in subgroups of patients with myalgic encephalomyelitis/chronic fatigue syndrome. Microbiome.

[14] Giloteaux L, Goodrich JK, Walters WA, Levine SM, Ley RE, Hanson MR (2016). Reduced diversity and altered composition of the gut microbiome in individuals with myalgic encephalomyelitis/chronic fatigue syndrome. Microbiome.

[15] Almeida C, Oliveira R, Soares R, Barata P (2020). Influence of gut microbiota dysbiosis on brain function: a systematic review. Porto Biomed J.

[16] Maiuolo J, Gliozzi M, Musolino V, Carresi C, Scarano F, Nucera S, Scicchitano M, Oppedisano F, Bosco F, Ruga S (2021). The contribution of gut microbiota-brain axis in the development of brain disorders. Front Neurosci.

[17] Mou Y, Du Y, Zhou L, Yue J, Hu X, Liu Y, Chen S, Lin X, Zhang G, Xiao H (2022). Gut microbiota interact with the brain through systemic chronic inflammation: implications on neuroinflammation, neurodegeneration, and aging. Front Immunol.

[18] Liu L, Wang H, Chen X, Zhang Y, Zhang H, Xie P (2023). Gut microbiota and its metabolites in depression: from pathogenesis to treatment. EBioMedicine.

[19] Guo C, Che X, Briese T, Ranjan A, Allicock O, Yates RA, Cheng A, March D, Hornig M, Komaroff AL (2023). Deficient butyrate-producing capacity in the gut microbiome is associated with bacterial network disturbances and fatigue symptoms in ME/CFS. Cell Host Microbe.

[20] Moher D, Liberati A, Tetzlaff J, Altman DG (2009). Preferred reporting items for systematic reviews and meta-analyses: the PRISMA statement. BMJ.

[21] Higgins JP, Thompson SG, Deeks JJ, Altman DG (2003). Measuring inconsistency in meta-analyses. BMJ.

[22] Menees S, Chey W (2018). The gut microbiome and irritable bowel syndrome. F1000Res.

[23] Andrioaie IM, Duhaniuc A, Nastase EV, Iancu LS, Luncă C, Trofin F, Anton-Păduraru DT, Dorneanu OS (2022). The role of the gut microbiome in psychiatric disorders. Microorganisms.

[24] Shan Y, Lee M, Chang EB (2022). The gut microbiome and inflammatory bowel diseases. Annu Rev Med.

[25] Radjabzadeh D, Bosch JA, Uitterlinden AG, Zwinderman AH, Ikram MA, van Meurs JBJ, Luik AI, Nieuwdorp M, Lok A, van Duijn CM (2022). Gut microbiome-wide association study of depressive symptoms. Nat Commun.

[26] Kers JG, Saccenti E (2022). The power of microbiome studies: some considerations on which alpha and beta metrics to use and how to report results. Front Microbiol.

[27] Gilbert JA, Blaser MJ, Caporaso JG, Jansson JK, Lynch SV, Knight R (2018). Current understanding of the human microbiome. Nat Med.

[28] Manor O, Dai CL, Kornilov SA, Smith B, Price ND, Lovejoy JC, Gibbons SM, Magis AT (2020). Health and disease markers correlate with gut microbiome composition across thousands of people. Nat Commun.

[29] Farhadfar N, Gharaibeh RZ, Dahl WJ, Mead L, Alabasi KM, Newsome R, IrizarryGatell V, Weaver MT, Al-Mansour Z, Jobin C (2021). Gut microbiota dysbiosis associated with persistent fatigue in hematopoietic cell transplantation survivors. Transplant Cell Ther.

[30] DeGruttola AK, Low D, Mizoguchi A, Mizoguchi E (2016). Current understanding of dysbiosis in disease in human and animal models. Inflamm Bowel Dis.

[31] Stanislawski MA, Dabelea D, Lange LA, Wagner BD, Lozupone CA (2019). Gut microbiota phenotypes of obesity. Npj Biofilms Microbiomes.

[32] Pisani A, Rausch P, Bang C, Ellul S, Tabone T, Cordina CM, Zahra G, Franke A, Ellul P (2022). Dysbiosis in the gut microbiota in patients with inflammatory bowel disease during remission. Microbiol Spectr.

[33] Li Z, Zhou J, Liang H, Ye L, Lan L, Lu F, Wang Q, Lei T, Yang X, Cui P (2022). Differences in alpha diversity of gut microbiota in neurological diseases. Front Neurosci.

[34] Wilmanski T, Diener C, Rappaport N, Patwardhan S, Wiedrick J, Lapidus J, Earls JC, Zimmer A, Glusman G, Robinson M (2021). Gut microbiome pattern reflects healthy ageing and predicts survival in humans. Nat Metab.

[35] Leite G, Pimentel M, Barlow GM, Chang C, Hosseini A, Wang J, Parodi G, Sedighi R, Rezaie A, Mathur R (2021). Age and the aging process significantly alter the small bowel microbiome. Cell Rep.

[36] Su X (2021). Elucidating the beta-diversity of the microbiome: from global alignment to local alignment. mSystems.

[37] Teofani A, Marafini I, Laudisi F, Pietrucci D, Salvatori S, Unida V, Biocca S, Monteleone G, Desideri A (2022). intestinal taxa abundance and diversity in inflammatory bowel disease patients: an analysis including covariates and confounders. Nutrients.

[38] Lin SW, Freedman ND, Shi J, Gail MH, Vogtmann E, Yu G, Klepac-Ceraj V, Paster BJ, Dye BA, Wang GQ (2015). Beta-diversity metrics of the upper digestive tract microbiome are associated with body mass index. Obesity.

[39] Wang T, Sternes PR, Guo X-K, Zhao H, Xu C, Xu H (2023). Autoimmune diseases exhibit shared alterations in the gut microbiota. Rheumatology.

[40] Suganya K, Koo BS (2020). Gut-Brain Axis: Role of Gut Microbiota on Neurological Disorders and How Probiotics/Prebiotics Beneficially Modulate Microbial and Immune Pathways to Improve Brain Functions. Int J Mol Sci.

[41] Visconti A, Le Roy CI, Rosa F, Rossi N, Martin TC, Mohney RP, Li W, de Rinaldis E, Bell JT, Venter JC (2019). Interplay between the human gut microbiome and host metabolism. Nat Commun.

[42] Lupo GFD, Rocchetti G, Lucini L, Lorusso L, Manara E, Bertelli M, Puglisi E, Capelli E (2021). Potential role of microbiome in chronic fatigue syndrome/myalgic encephalomyelits (CFS/ME). Sci Rep.

[43] Armstrong CW, McGregor NR, Lewis DP, Butt HL, Gooley PR (2016). The association of fecal microbiota and fecal, blood serum and urine metabolites in myalgic encephalomyelitis/chronic fatigue syndrome. Metabolomics.

[44] Johnson EL, Heaver SL, Waters JL, Kim BI, Bretin A, Goodman AL, Gewirtz AT, Worgall TS, Ley RE (2020). Sphingolipids produced by gut bacteria enter host metabolic pathways impacting ceramide levels. Nat Commun.

[45] La Fata G, Weber P, Mohajeri MH (2014). Effects of vitamin E on cognitive performance during ageing and in Alzheimer's disease. Nutrients.

[46] Huang AA, Huang SY (2023). Quantification of the effect of vitamin e intake on depressive symptoms in united states adults using restricted cubic splines. Current Develop Nutr.

[47] Ciarcià G, Bianchi S, Tomasello B, Acquaviva R, Malfa GA, Naletova I, La Mantia A, Di Giacomo C (2022). Vitamin E and non-communicable diseases: a review. Biomedicines.

[48] Gothandapani D, Makpol S (2023). Effects of vitamin E on the gut microbiome in ageing and its relationship with age-related diseases: a review of the current literature. In Int J Mol Sci.

[49] Gao K, Mu CL, Farzi A, Zhu WY (2020). Tryptophan metabolism: a link between the gut microbiota and brain. Adv Nutr.

[50] Lee JS, Kang JY, Park SY, Hwang SJ, Bae SJ, Son CG (2024). Central 5-HTergic hyperactivity induces myalgic encephalomyelitis/chronic fatigue syndrome (ME/CFS)-like pathophysiology. J Transl Med.

[51] Navaneetharaja N, Griffiths V, Wileman T, Carding SR (2016). A role for the intestinal microbiota and virome in myalgic encephalomyelitis/chronic fatigue syndrome (ME/CFS)?. J Clin Med.

[52] Roman P, Carrillo-Trabalon F, Sanchez-Labraca N, Canadas F, Estevez AF, Cardona D (2018). Are probiotic treatments useful on fibromyalgia syndrome or chronic fatigue syndrome patients?. Syst Rev Benef Microbes.

[53] Ser HL, Letchumanan V, Goh BH, Wong SH, Lee LH (2021). The Use of fecal microbiome transplant in treating human diseases: too early for poop?. Front Microbiol.

[54] Lee C, Louie T, Bancke L, Guthmueller B, Harvey A, Feuerstadt P, Khanna S, Orenstein R, Dubberke ER (2023). Safety of fecal microbiota, live-jslm (REBYOTA™) in individuals with recurrent clostridioides difficile infection: data from five prospective clinical trials. Ther Adv Gastroenterol.

[55] Jain N, Umar TP, Fahner AF, Gibietis V (2023). Advancing therapeutics for recurrent clostridioides difficile infections: an overview of vowst's FDA approval and implications. Gut Microbes.

[56] Salonen T, Jokinen E, Satokari R, Lahtinen P (2023). Randomized, double-blinded, placebo-controlled pilot study: efficacy of faecal microbiota transplantation on chronic fatigue syndrome. J Transl Med.

[57] Lacerda EM, Geraghty K, Kingdon CC, Palla L, Nacul L (2019). A logistic regression analysis of risk factors in ME/CFS pathogenesis. BMC Neurol.

[58] Fransen F, van Beek AA, Borghuis T, Meijer B, Hugenholtz F, van der Gaast-de Jongh C, Savelkoul HF, de Jonge MI, Faas MM, Boekschoten MV (2017). The impact of gut microbiota on gender-specific differences in immunity. Front Immunol.

