# Supplementary material for: Clinical evidence of the link between gut microbiome and myalgic encephalomyelitis/chronic fatigue syndrome: a retrospective review
Source: Eur J Med Res. 2024 Mar 1;29:148. doi: 10.1186/s40001-024-01747-1 (PMC10908121; doi:10.1186/s40001-024-01747-1)
Supplement: Supplementary file 1 — Additional file 1: Table S1. List of selected 11 clinical studies in the present review. [file 40001_2024_1747_MOESM1_ESM.docx]

**Table S1. List of selected 11 clinical studies in the present review**

| **No.** | **Reference**  **(PMID)** | **Country** | **Participants** | **Main findings** |
| --- | --- | --- | --- | --- |
| I | Guo  2023  (36758522) | USA | 106 CFS;  91 Healthy control | 1. ME/CFS patients display significant dysbiosis in their gut microbiome. 2. Bacterial abundances, functions, and SCFAs differ in ME/CFS compared to healthy control. 3. The decreased presence of *F. prausnitzii* and *E. rectale* in ME/CFS may lead to butyrate deficiency. 4. Low *F. prausnitzii* abundance is associated with more severe fatigue symptoms in ME/CFS. |
| II | Xiong  2023  (36758521) | USA | 149 CFS;  79 Healthy control | 1. Multi-omics approaches identified phenotypic, gut microbial, and metabolic biomarkers for ME/CFS. 2. ME/CFS is associated with reduced gut microbial diversity and increased plasma sphingomyelins. 3. Short-term ME/CFS exhibits more severe gut microbial dysbiosis, including decreased butyrate levels. 4. Long-term ME/CFS demonstrates more significant metabolic and clinical aberrations |
| III | Lupo  2021  (33782445) | Italy | 35 CFS;  70 Healthy control | 1. In ME/CFS, stool samples show reduced the abundance of Anaerostipes and increased *Phascolarctobacterium*. 2. ME/CFS exhibits elevated levels of glutamic acid and argininosuccinic acid, along with decreased levels of alpha-tocopherol in stool. |
| IV | Kitami  2020  (33199820) | Japan | 48 CFS;  52 Healthy control | 1. Lipoprotein and microbiome profiles are strongly associated with sleep disruption, featuring distinct markers for cognitive parameters. 2. Changes in sleep, lipoproteins, and microbiomes manifest early in the course of the illness, suggesting potential biomarker applications in larger cohorts. 3. Notably, molecular changes related to sleep clustering in ME/CFS are prominent within the Japanese population. |
| V | Mandarano 2018  (29375937) | USA | 49 CFS;  39 Healthy control | 1. Specific eukaryotic taxa are not identified in individuals with ME/CFS. 2. There is a no difference of eukaryotic diversity between ME/CFS and healthy individuals. 3. A non-significant increase is observed in the ratio of fungal phyla Basidiomycota to Ascomycota in ME/CFS patients. |
| VI | Nagy-Szakal 2017  (28441964) | USA | 50 CFS;  50 Healthy control | 1. Excessive short-chain fatty acids (SCFAs) by gut microbiome in ME/CFS has detrimental effects on their energy metabolism |
| VII | Amstrong 2017  (N/A*) | Australia | 34 CFS;  25 Healthy control | 1. The heightened production of SCFAs through microbial fermentation in the gut of ME/CFS patients may be linked to adverse effects on the host's energy metabolism. |
| VIII | Giloteaux 2016 ^a^  (27721367) | USA | 1 CFS;  1 Healthy control (Monozygotic twins ) | 1. ME/CFS patients exhibit reduced diversity in both the gut microbiome and virome, characterized by a decreased presence of beneficial bacteria such as Faecalibacterium and Bifidobacterium. 2. There is an elevated presence of tailed dsDNA Caudovirales bacteriophages |
| IX | Giloteaux 2016 ^b^  (27338587) | USA | 49 CFS;  39 Healthy control | 1. Damage to the gut mucosa leads to increase of microbial translocation in ME/CFS, potentially causing alterations in antimicrobial regulators and dysregulation of the innate immune system. 2. ME/CFS is associated with a less diverse and unstable community of gut microbiota. 3. There is no single specific alteration of the gut microbiota common to all ME/CFS patients. 4. Gut dysbiosis may contribute to the severity of some symptoms in ME/CFS. |
| X | Shukla  2015  (26683192) | USA | 10 CFS;  10 Healthy control | 1. The systemic impact of a modified gut microbiome in ME/CFS patients. 2. During an exercise challenge, significant alterations in the abundance of major bacterial phyla in the gut were observed in ME/CFS patients but not in healthy controls. 3. ME/CFS patients experienced a delay in the clearance of bacteria from their bloodstream. |
| XI | Frémont  2013  (23791918) | Belgium | Belgian:  18 CFS;  19 Healthy control | 1. ME/CFS leads to alterations in the intestinal microbiota. 2. The composition of the gut microbiota differed between Belgian controls and Norwegian controls. 3. A highly significant distinction was observed between Norwegian controls and Norwegian patients. 4. Patients exhibited increased proportions of Lactonifactor and Alistipes, along with a reduction in several Firmicutes populations. |
|  |  |  | Norwegian:  25 CFS;  17 Healthy control |  |

* https://link.springer.com/article/10.1007/s11306-016-1145-z, N/A: not available.
