# Supplementary material for: Clinical evidence of the link between gut microbiome and myalgic encephalomyelitis/chronic fatigue syndrome: a retrospective review
Source: Eur J Med Res. 2024 Mar 1;29:148. doi: 10.1186/s40001-024-01747-1 (PMC10908121; doi:10.1186/s40001-024-01747-1)

**Figure S1 Risk of bias summary**

The Cochrane risk of bias tool was used to evaluate the risk of bias for each trial. Green-colored symbol corresponds to low risk of brisk of bias assessment, yellow corresponds to unclear risk of bias, and red corresponds to high risk of bias.


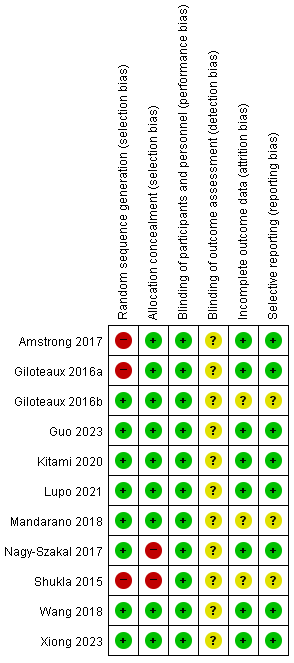

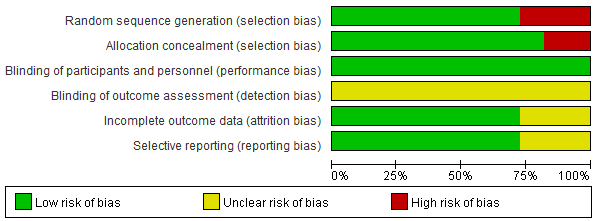

Supplement: Supplementary file 2 — Additional file 2: Figure S1. Risk of bias summary. [file 40001_2024_1747_MOESM2_ESM.docx]
