# Supplementary material for: Clinical evidence of the link between gut microbiome and myalgic encephalomyelitis/chronic fatigue syndrome: a retrospective review
Source: Eur J Med Res. 2024 Mar 1;29:148. doi: 10.1186/s40001-024-01747-1 (PMC10908121; doi:10.1186/s40001-024-01747-1)

**Figure S2 Funnel plot of included 6 studies for meta-analyzing gut microbiome α-diversity**

SE: Standardized Error for SMD; SMD: standard Mean Difference.


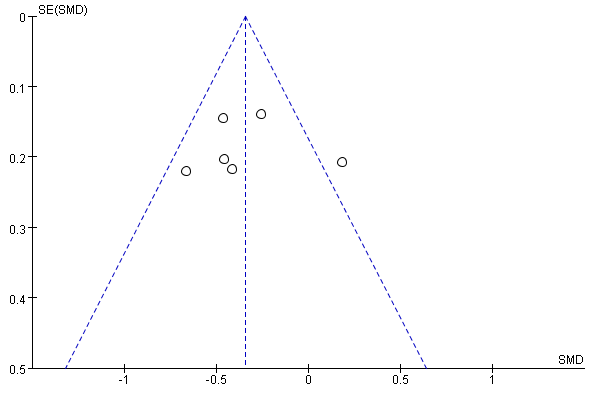

Supplement: Supplementary file 3 — Additional file 3: Figure S2. Funnel plot of included 6 studies for meta-analyzing gut microbiome α-diversity. [file 40001_2024_1747_MOESM3_ESM.docx]
